# The relationality of parts for narrative identity constitution in the corporate profile translations of China’s multinational corporations

**DOI:** 10.1057/s41599-023-01603-3

**Published:** 2023-03-14

**Authors:** Li Wang, Lay Hoon Ang, Fumeng Gao, Hazlina Abdul Halim

**Affiliations:** 1 School of Foreign Languages, Fuyang Normal University, Fuyang, China; 2 Department of Foreign Languages, Faculty of Modern Languages and Communication, Universiti Putra Malaysia, Selangor, Malaysia

**Keywords:** Language and linguistics, Business and management

## Abstract

The corporate profile translations of multinational corporations (MNCs) in emerging economies such as China possess rich information for narrative analysis. Nevertheless, how the parts of a corporate profile translation form a whole narrative remains undertheorized. This study, therefore, examines the relationality of parts in the corporate profile translations of China’s MNCs by integrating William Labov’s narrative structure with Margaret Somers’ narrative identity theory. Specifically, we conduct a theoretical thematic analysis of how constituents form a whole narrative in relevant corporate profiles, of the shifts in the relationality of parts from the Chinese source texts (STs) to the English target texts (TTs) of these profiles, and of the influences of these shifts on the constitution of corporate identities in the target texts. Our results show that in the corporate profiles of Chinese MNCs, episodes are not randomly selected and related to each other but follow predominant patterns. However, we find no unified patterns in the shifts in the relationality of parts via the corporate profile translation of China’s MNCs. We thus reveal how corporations’ identities are constituted in diverse ways that reflect their fluid and unique features. Accordingly, our findings have implications for translation studies and corporate communications.

## Introduction

Emerging economies such as China have benefited from globalization (Erixon, 2018; Progunova et al., 2019). Specifically, the globalization of China’s corporations constitutes ‘an unprecedented phenomenon in corporate history’ (David and Hung, 2020, p. 8). However, there has never been a smooth path for China to expand its markets globally. The financial crash in 2008, the US–China trade war in 2018, and the COVID-19 pandemic constitute three ‘body blows’ that have damaged the open system of trade and impeded globalization (The Economist, 2020). In addition, Western consumers tend to have a ‘poorer’ image of emerging markets (Held and Bader, 2018, p. 10). Thus, to address this changing global social and economic situation while responding to the Chinese government’s ‘Go Global’ strategy (Morrison, 2019) and prioritizing their development needs, China’s multinational corporations (MNCs) have updated their ways of ‘storytelling’ via corporate profile translation to constitute corporate identities for overseas markets (Xinhua, 2018). These translations are, therefore, rich sources for relevant research.

The literature has defined the mode of storytelling in international and business communications as narrative. Narrative research in the social sciences has provided the theoretical foundation for this interpretation (Denning, 2006; Hagström and Gustafsson, 2019; Zhi, 2019; Zhao, 2017). Furthermore, Margaret Somers has advocated using the relational approach in narrative studies, defining narrativity features as part of a systematic whole (Somers and Gibson, 1993; Somers, 2008). Specifically, Somers proposes that since the connected parts of narratives are ‘embedded in time and space, constituted by causal emplotment’ (Somers and Gibson, 1993, p. 27), they form a relational network. Identities are constituted within this relational network. Thus, the analysis unit for narrative study should ‘ultimately [be] an entire narrative’ (Baker, 2010, p. 349). Somers’ idea of the relationality of parts in a narrative was first introduced to the translation literature by Baker (2006), who has argued that ‘language users, including translators and interpreters’ exploit narrativity features in the translating process (Baker, 2007).

MNCs’ corporate profiles and their translations are prominent in their stories, which constitute their corporate identities for target markets among all their possible means of corporate communication. There are two reasons for the ‘prominence’ of corporate profiles. First, they convey information about a corporation that is selected and linked to fulfill a ‘self-presentation function’ (Capriotti and Moreno, 2007, p. 88). A standard corporate profile represents the professional introduction of a corporation describing its business information, general business activities, company strategy, scope of work, financial considerations, etc. (Capriotti and Moreno, 2007; Richa, 2014). Second, because corporate profiles are ‘the only areas for which a suitable combination of the communication resources available is used’ (Capriotti and Moreno, 2007, p. 89), they function as the most direct way for target readers to acquire a powerful impression of a corporation (Richa, 2014). Effective examples of Chinese MNCs’ corporate profiles include those of the state-owned China Huaneng Group and the Power Construction Corporation of China as well as those of certain private corporations, e.g., Huawei and the Zhejiang Geely Holding Group. Additionally, some corporations have attempted to create unconventional corporate profiles and translations when presenting their identities, e.g., the Ruyi Group. Ruyi has crafted brief but artistically presented Chinese and English corporate profiles (China Ruyi, n.d.) that are congruent with its ambition to become a top ‘luxury fashion group’ rather than remaining a simple ‘textile manufacturer’ (Zhu and Ju, 2018).

Bruner and Hodges categorize narratives using different ways of constructing reality into discrete genres (Bruner, 1991; Hodges, 2011). Furthermore, Mona Baker has defined translation as ‘a genre in its own right’ (Baker, 2018). However, storytelling in corporate profile translation, as a genre, ‘has received inadequate attention in [the] mainstream [translation] literature’ (Yu and Liu, 2018, p. 348), especially in research on corporations in emerging economies. Previous translation studies that have used the narrative perspective have mainly empirically analysed examples drawn from the literature, social services organizations, or various media outlets (Constantinou, 2017; Corman, 2016; Jacobs, 2018; Jacobs and Maryns, 2021; Zhao et al., 2022). There is also abundant research on the individual components of narratives involving rhetorical analysis (Dodge and Keränen, 2018), interview analysis (Johnson, 2016), corpus analysis (Rizzo, 2018), linguistic analysis (Gunderson, 2016), or process tracing (Bolton and Minor, 2016).

In contrast, the systematic literature on narratives in the genre of corporate profile translation remains undertheorized. Moreover, due to the interdisciplinary nature of narrative study, it is unclear whether the methods used to analyse narratives in one genre can be adopted to those in another. To address this gap, this research addresses the following research questions: How do the parts relate to form a whole narrative in the corporate profiles of China’s MNCs? How is the relationality of parts in corporate profiles shifted during their translation? Furthermore, to what extent do these shifts influence the constitution of corporate identities?

## Theoretical background

### Relational approach in narrative studies

Narratives, in general, are ‘storied ways of knowing and communicating’ (Riessman, 2005, p. 2) that not only represent but also (re)produce reality (Mura, 2015, p. 226). However, while an infinite variety of events take place every day, not all of them are significant enough for a narrator to form a relevant narrative. The ‘storied form’ of a narrative is what distinguishes it from the random events occurring around us (Hansen et al., 2017; Scheibe and Barrett, 2017). Narrators thus attempt to assemble or integrate certain events into narratives (Somers and Gibson, 1993, p. 3). These assembled or integrated events are connected, forming different relationships. Through the ways narrators locate themselves or are located in these relationships, meanings are discerned (Somers, 1994, p. 616). In other words, ‘identity formation takes place within these relational settings’ (Somers, 1994, p. 626).

With this relational approach, Somers (1994) proposes a framework of narrativity features that are ‘defining’ and ‘interdependent’ when ‘constituting narrative’ (Baker, 2006, 2007). She suggests that narratives function in a network where time, space, and analytical relationality construct reality. In this network, four features are applicable when evaluating narratives with the social science approach: the relationality of parts, causal emplotment, selective appropriation, and temporality. These four features function as an organic whole, reflecting how ‘narratives are constellations of relationships (connected parts) embedded in time and space, constituted by causal emplotment’ (Somers, 1994, p. 616). Somers primarily bases her framework on social movement organizations’ monolingual narratives. However, in terms of an organization’s narratives in different languages, for example, its corporate communication translation, more attempts must be made to embed this framework within multilingual contexts. In addition, Mona Baker compares Somers’ four narrativity features to the ten narrativity features of another theorist, Jerome Bruner (Baker, 2006). She observes overlaps among these two scholars’ definitions of narrativity features, despite their disparate fields. For example, Somers’ relationality of parts is regarded as equivalent to Bruner’s hermeneutic composability in his narrativity framework (Baker, 2006; Sinalo, 2020; Stojanovic, 2014). Hence, in *Narratives in and of Translation* (Baker, 2005), Baker defines Somers’ narrativity features as the four core features of narrativity before introducing them into translation research. Nevertheless, Baker does not discuss narrative identity constitution within the relational setting of translation studies.

### Relationality of parts

Among these four features, relationality is ‘a founding principle for identity formation and transition’ (Hollway, 2010, p. 216). However, while Somers proposes the relationality of parts within a narrative, she does not provide a clear definition for it. Indeed, in some of her works, Margaret Somers even describes this feature with another name, the ‘connectivity of parts’ (Somers and Gibson, 1993; Somers, 1992, 1994). Mona Baker has thus suggested that this relationality/connectivity is simply how the ‘individual elements…are configured within a narrative…[to] acquire meaning’ (Baker, 2018). In other words, according to Baker, the relationality of parts refers to the relationship between the whole narrative and its parts.

However, regarding what these parts actually are, scholars express diverse views. Mona Baker claims that the individual elements of a narrative, i.e., its parts, include ‘events, characters, linguistic items, layout, imagery, etc.’ (Baker, 2014, p. 168). Nevertheless, she does not explain how these various elements work together to form a whole narrative. Bamberg and Marchman, on the other hand, define episodes as the elements holding a narrative together (Bamberg and Marchman, 1990, 1991; Mandler, 2014). This notion that episodes are the parts composing a narrative is congruent with Margaret Somers’ theory; she regards the relationality of parts as the transformation of events into episodes in a narrative (Somers, 1994). Therefore, in this study, we employ Bamberg and Marchman’s definition of episodes while analysing the relationality of parts in a narrative.

Furthermore, since episodes are selected and sequenced events, it is essential to know the boundary of an event, especially in regard to an event selected for a corporate profile. In traditional narrative studies, scholars have determined events according to their contribution to a literary work. However, Tilmann Köppe has proposed that an event simply occurs whenever ‘something happens’ (Köppe, 2014, p. 102), despite its literary significance. Specifically, he argues that an event requires three designations: first, it should designate a substance; second, it should designate what is true for this substance; third, it must designate ‘the interval or point in time at which what is said about the substance is true’ (Köppe, 2014, p. 102). However, Köppe admits that the boundaries of events are approximate. He has therefore suggested quoting an event’s ‘linguistic means’, such as a complete and single sentence, to delineate it (Köppe, 2014). For instance, the following sentences from a corporate profile clearly designate events: ‘Since 2010, ZTE has ranked among the world’s Top-5 patent application firms under the Patent Cooperation Treaty (PCT) each year according to the World Intellectual Property Organization’; ‘ZTE Corporation is a global leader in telecommunications and information technology’ (ZTE, 2018). As such, Köppe has extended the traditional conceptual boundaries of events in literary studies to the boundaries of all event types, literary or nonliterary.

After defining the episodes that constitute discrete parts, it is essential to identify how they are organized into a whole. Only when they are connected meaningfully can this whole be regarded as a narrative (Köppe, 2014). According to Somers and Gibson, two issues drive the interrelationship between the parts and a whole. First, the organization of a narrative’s parts must be ‘around a central plot’, i.e., they must be ‘structurally organized’ (Hodges, 2011, p. 42). Moreover, episodes must be tellable because relationality accounts for why ‘events’ are turned into ‘episodes’—the components of a narrative (Somers and Gibson, 1993). To further illustrate the relationality of parts through empirical research, scholars such as Bruner and Hodges have cited Labov’s account of narrative structure (Bruner, 1991; Hodges, 2011). Specifically, in Hodges’ seminal empirical study, he employs Labov’s structure to analyse hermeneutic composability (the relationality of parts) in political speeches on terrorism. Hence, his research has been touted as ‘well-designed’, ‘well-executed’, and ‘multidisciplinary’ in the literature on dynamic narrative connections (Dunmire, 2012, p. 632).

Labov’s narrative structure includes two key points: ‘the irreducible clausal sequences’ that explain how episodes are organized and ‘the evaluation of events’ that justify the tellability of episodes (as cited by Bruner, 1991, p. 12). These two points resolve the abovementioned issues concerning Somers’ relationality of parts. That is, narrative structure is based on a precipitating event that functions as both the starting point and lead of a thread and ‘the final section’, the ‘coda’, of a narrative (Hodges, 2011, p. 58). A precipitating event thus triggers storytelling. On the other hand, a coda may mark the end of a narrative, illustrate its events’ effect on the narrator, or bridge the gap between the time at the end of a narrative and the reader’s present (Labov, 1973). The precipitating event and coda of the English corporate profile of CRRC Corporation Limited (CRRC) are listed below as examples.

Example 1:

Headquartered in Beijing, CRRC Corporation Limited (CRRC) has 46 wholly-owned and majority-owned subsidiaries with over 170,000 employees (CRRC, n.d.).

Example 1 is the precipitating event of the profile. It is placed at the beginning of the profile, introducing the corporation’s basic information, such as its location and size, as the starting point for presenting the corporation to its stakeholders. Following the precipitating event, the profile introduces the corporation’s more complex information, including its business scope, present achievements, and future mission and strategies. Finally, the narrative flows towards its coda, which is shown in Example 2.

Example 2:

Joining hands with you, our dear customers and friends, CRRC will find the most effective solutions for the sustainable development of railway transportation (CRRC, n.d.).

The coda thus brings the narrative to an end. It directly addresses its readers, calling for cooperation with them. In this way, the gap between the previous episodes and the readers is bridged.

From a narrative’s starting point to its coda, episodes describing complicating actions are interwoven with its main thread via different tools, whereby evaluations of these episodes warrant their tellability (Labov, 1973). In addition, two types of evaluations initially proposed by Labov (1973) are identified in Hodges’ empirical study of political speeches (Hodges, 2011): external evaluations and embedded evaluations. The former are the remarks narrators use to address their readers when they cease their narrating action. The latter are the descriptions ‘embedded within the complicating actions’ (Hodges, 2011, p. 43) that do not impede narration. Below, we provide some examples of external evaluation and embedded evaluation from corporate profiles.

Example 3:

The high-speed trains manufactured by CRRC have become one of the jewels in China’s crown to showcase China’s development achievements to the world. (CRRC, n.d.).

Example 3 is an external evaluation taken from the corporate profile of CRRC. It is positioned in the middle of a text beneath a long list of the corporation’s products to showcase the significance of its main product. After this single-sentence evaluation, the profile introduces another episode, the corporation’s mission.

Example 4:

CRRC will *vigorously* implement the strategy for internationalization, diversification and collaborative development, and strive to be the world’s *leading* provider of high-end equipment system solutions (CRRC, n.d.).

Example 4 is an embedded evaluation taken from the profile of CRRC. While describing this corporation’s development strategy, the narrator describes how ‘vigorously’ this implementation is being pursued, emphasized by the underlined text. Accordingly, the narrator describes the aim of this strategy, to be the ‘world’s leading provider’ (CRRC, n.d.). Such an evaluation highlights the strength of this strategy.

By justifying tellability, evaluations express the critical point of a narrative from its speaker’s perspective (Andrews et al. 2013, p. 32). Notably, evaluation is not static in storytelling. It is an interaction between speaker and listener, a constantly shifting ‘marker [of] a speaker’s presentation of self’ (Gwyn, 2000, p. 316). Hence, in translation studies, shifts in evaluations from source text (ST) to target text (TT) indicate changes in the markers that corporations present to their target readers.

Using the translation shift in CRRC’s profile’s external evaluation as an example once again, the ST and TT of this evaluation are listed below, together with a back translation for language clarity.

Example 5:

Source Text: 中国中车制造的高速动车组系列产品, 已经成为中国向世界展示发展成就的重要名片 (CRRC, n.d.)。

Back Translation: The high-speed trains manufactured by CRRC have become *an important business card*, to showcase China’s development achievements to the world.

Target Text: The high-speed trains manufactured by CRRC have become *one of the jewels in China’s crown*, to showcase China’s development achievements to the world (CRRC, n.d.).

This example includes an evident shift when translating the external evaluation. The narrator evaluates CRRC’s high-speed trains product as ‘an important business card’ in the Chinese ST. Such an evaluation has been upgraded to ‘one of the jewels in China’s crown’ via the translation into the English TT (CRRC, n.d.). Hence, CRRC’s identity has been repositioned from a manufacturing corporation doing business abroad to a corporation adding glory and honor to its home country.

That is, the relationality of parts refers to how precipitating events, complicating actions, codas and evaluations work together to form a narrative whole. Accordingly, the relational network of a narrative is where narrative identities are embedded.

**Fig. 1:**
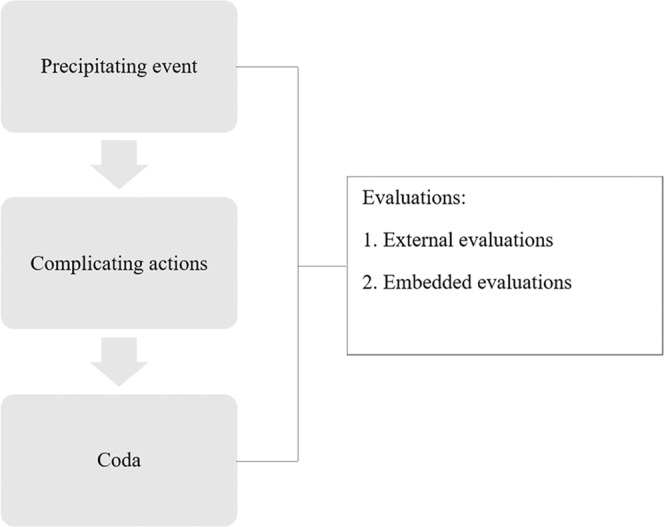
Relationality of parts (Hodges, 2011; Labov, 2006; Somers, 1994). The precipitating event, the complicating actions, the coda, and the evaluations work together to form a narrative whole.

This framework of the relationality of parts is briefly illustrated in Fig. 1 below.

### Constitution of identities

As mentioned above, identities are constituted within the relational settings of a narrative (Somers, 1992, p. 600). As it is based on the notion of ‘identity’, e.g., individual, social or organizational identity (Cornelissen et al., 2007), corporate identity is also closely related to the relationality of parts within a narrative.

More critically, corporate identity refers to ‘the presentation of an organization to every stakeholder’ (Melewar and Karaosmanoglu, 2006, p. 864). Organizations thus express ‘their own individuality and uniqueness’ through their corporate identity (Cornelissen et al., 2007, p. S7), allowing them to differentiate themselves. Cornelissen et al. (2007, p. S8) emphasize that such collective identities tend to be ‘fluid rather than fixed’. This is especially true for a corporate identity, which must ‘respond to dynamic changes in the external world’ (Grant, 1999). Collectively, these two features of corporate identities—fluidity and uniqueness—have allowed corporations to possess a unique corporate identity, which changes over time and space.

Addressing the unique and fluid characteristics of identities through a narrative approach Somers (1994) states that identities should never be regarded as rigid categorizations. Indeed, she proposes avoiding ‘categorical rigidities by emphasizing the embeddedness of identity in overlapping networks of relations that shift over time and space’ (Somers, 1994, p. 607). She elaborates that narrative identity is constituted within ‘relational settings of contested but patterned relations among narratives, people, and institutions’ (Somers, 1994, p. 626). According to Somers, a relational setting is thus ‘a pattern of relationships among institutions, public narratives, and social practices’ (Somers, 1994, p. 626). As such, identity is not a state but a position within a relational setting. Hence, examining the relationships of the various parts within a setting can help locate an identity. Studying the shifts among these relationships will therefore facilitate clarifying the changes in the constitution of an identity.

In this study, we define episodes as a narrative’s parts that are organized along the path from precipitating event to coda and justified by external or embedded evaluations. Additionally, we compare the organization of the episodes in STs and TTs to determine if there are any shifts and, when such shifts exist, their influences on identity constitution. However, corporate profile translations comprise a distinct genre relative to the literary works and political speeches used in previous research. They contain selected and seemingly independent episodes—origins, business types, strategies, and corporate social responsibilities (CSRs)—that provide target readers with the necessary information about a corporation. Hence, throughout this study, we remain open to any new information that may emerge in the coding process concerning the relationality of parts in corporate profiles and their translation.

## Methods

In this study, we use a qualitative approach with homogenous purposeful sampling. Two causes may trigger a shift in the self-presentation of a corporation: environmental forces and identity misalignment (Abratt and Mingione, 2017). Homogenous purposeful sampling can therefore avoid confounding the elements in environmental forces and help researchers focus on shifts in identities within focal settings. We selected 12 state-owned Chinese manufacturing multinational corporations as our sample. These samples were identified via an annual list of MNCs jointly certified as Chinese multinational corporations by the official China Enterprise Confederation (CEC) and China Enterprise Director Association (CEDA) (CEC and CEDA, 2020). Additionally, we ensured that all the selected corporations were state-owned manufacturing corporations by reviewing their registered information on Qichacha, a Chinese business intelligence company that provides ‘business data, credit information, and analytics on private and public companies’ (Crunchbase, n.d.). As they are state-owned, all Chinese multinational corporations function in a similar political and social context (Leutert, 2020). In addition, we purposefully selected corporations in the manufacturing industry due to their rich information for qualitative research on corporate communications. Chinese multinational manufacturing corporations are not only part of the ‘largest industry in China’ (Dalci, 2018) but are also part of a major emerging economy (Li, 2018). Since it is emerging, this economy offers ‘an especially salient environment’ for exploring organizational identity formation (Clegg et al., 2007). Below, Table 1 lists the industrial composition of the twelve samples. As manufacturing corporations, they are categorized according to their ownership and industrial materials (Goto and Sueyoshi, 2020).

**Table 1:** Industry composition of the samples.

Ownership	Type of industry	Number of companies
State-owned	Chemical	3
	Nonferrous metal and metal products	4
	Machinery	2
	Transportation equipment	1
	Electrical equipment	1
	Pharmaceutical products	1

Moreover, we employ theoretical thematic analysis to ‘identif[y], analys[e] and repor[t] the patterns (themes) within the data’ during coding and data analysis (Braun and Clarke, 2006, p. 79). The unique feature of theoretical thematic analysis is that it extracts themes and analyses patterns to answer specific research questions (Braun and Clarke, 2006; Maguire and Delahunt, 2017). In this research, episodes are thus coded and analysed according to our three research questions as follows:

Our first step in analysing the relationality of parts is determining the parts and the structure of each narrative in the samples. On the one hand, we regard episodes as the parts of a narrative. Two additional issues clarify how these parts form a whole narrative: how episodes transpire from the precipitating event, through complicating actions, to the coda; and how episodes are justified as tellable via interwoven evaluations (Bruner, 1991; Hodges, 2011; Labov, 1973). Hence, three categories of themes are initially extracted: precipitating event, complicating action, and coda. Evaluations are coded and extracted from the episodes and then defined as either external to or embedded in their text. This clarifies the patterns of the relationality of parts in the samples, answering the first research question. Second, differences and similarities regarding the relationality of parts between source and target texts are identified to determine if, via translation, shifts occur. Last, shifts in the relational settings between source and target texts are examined to determine if and how the constitution of identities within these settings has shifted.

To perform triangulation and validate the data analysis results, the researchers conducted several semi-structured in-depth interviews. Four translators and one translation project manager who had participated in corporation profile translation among Chinese multinational corporations were interviewed using pseudonyms. The aim of the survey was to verify the shifts in relationality of parts from ST to TT and the decisions concerning corporate identities behind these shifts. The researchers designed three core questions related to the research questions:

1. What does the corporate profile translation process involve?
2. What requirements have you received from a corporation for translating its corporate profile?
3. What does a corporation want to show its target stakeholders in a corporate profile translation?

Each interview took 15–20 min via telephone or in an online meeting, as required by the interviewee. These interviews were recorded and transcribed in a written form. Afterward, the researchers analysed the transcripts with thematic analysis. These findings of which were cross-checked with the corporate profiles’ data analysis results, i.e., the translation processes behind the translation shifts in the relationality of parts.

### Data analysis

Since the relationality of parts concerns the formation of a whole narrative, it cannot be determined without viewing all the parts systematically. Thus, below, we identify patterns in the relationality of parts by examining how the precipitating events, complicating actions, codas, and evaluations work together in the samples. Consequently, we reveal two patterns of the relationality of parts in our data analysis. One is temporally sequenced, the other is causally sequenced. No other patterns than these two are detected. Moreover, we find that the distribution of these two patterns among our sample texts is not even.

**Fig. 2:**
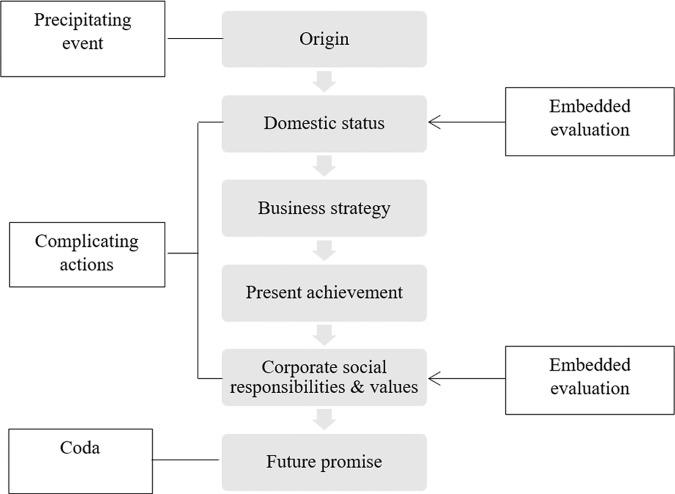
Relationality of parts in the ST of Sample 3. Sample 3 is an example of the temporally sequenced pattern starting from the corporation’s origin and ending with its future promise.

The first pattern occurs in seven of the twenty-four (twelve pairs of) sampled texts, a minor part of all the samples that includes the STs of Samples 1, 2, and 9 and both the STs and TTs of Samples 3 and 4. This pattern involves building a narrative with episodes via a central plot that follows a temporal sequence, although we observe slight variances among the samples. Samples within this pattern usually start their narration with the firm’s origin as the precipitating event and then present its achievements or future promise as the coda. This narration moves chronologically, and its complicating actions comprise the corporation’s achievements and business operations. Embedded evaluations are interwoven with this central thread. Using the ST of Sample 3 as an example, Fig. 2 illustrates how episodes are organized in the pattern.

Sample 3 is the ST of the corporate profile of ChemChina, which opens with its founding date and concludes with its future promise, as shown in Fig. 2. Abundant as its information is, this profile is not loosely written; it develops a description of how the corporation is growing and progressing and how it plans to shoulder more responsibilities in the future. Moreover, there are evaluations highlighting the corporation’s elevated domestic status and strong sense of corporate social responsibility, e.g., it has the competence and strong will needed to fulfill the future promise it describes at the end of its story. Thus, this corporation’s dynamic identity is narrated by presenting this identity in multiple instances chronologically.

Instead of being sequenced temporally, the second pattern provides clear signs that indicate the causal relationships among episodes. This pattern informs a major proportion of the samples: seventeen of the twenty-four (twelve pairs of) sampled texts. In regards to detecting causal relationships, Dahlstrom (2013) proposes that events are causal as long as each of them is the direct cause of a later event (Dahlstrom, 2013). Specifically, this model is based on the logical criterion of necessity; that is, element ‘A’ is considered causally related to element ‘B’ if element ‘B’ could not occur in the narrative without element ‘A’ (Dahlstrom, 2010). Accordingly, we identify the causal relationships among the episodes in our samples via this causal network model.

**Fig. 3:**
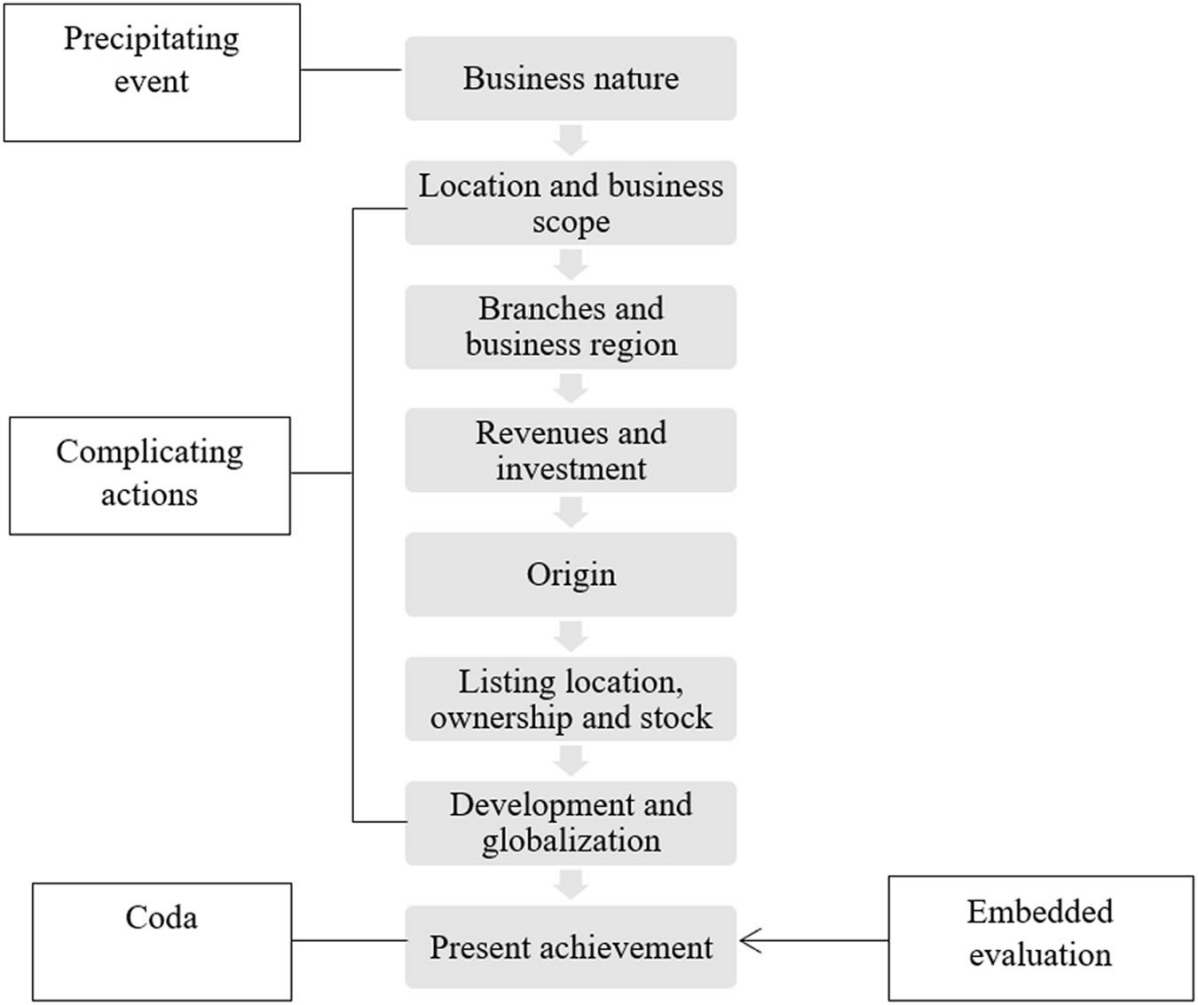
Relationality of parts in the TT of Sample 5. Sample 5 is an example of the causally patterned pattern starting from the corporation’s business nature and ending with its present achievement.

Samples falling into this pattern thus utilize causal sequencing—a corporation’s nature of business is the narrative’s precipitating event, its qualifications are the complicating actions, and its achievement is the coda. Below, Fig. 3 illustrates the relationality of parts in an example from Sample 5’s TT. This text begins by stating the business nature of the corporation and ends with an episode concerning its present achievement; the only evaluation is embedded in its description of this achievement. The complicating actions between the two ends of this kind of narrative progress from a statement of the corporation’s basic information and qualifications to a description of its development. Therefore, as the triggering event is the corporation’s business nature while the coda and focal point describe its present achievement, the corporation presents its story in a causal sequence. That is, the corporation’s nature, qualifications, and other basic aspects lead to its success, the focus of its narrative. In other words, its corporate identity is accumulated through its efforts in all aspects, which is also neither static nor rigid.

Having detected the patterns in the relationality of parts among all the samples, we next explore whether the relationality of parts shifts in the corporate profiles of all these corporations within their translations for different target readers. Our results show that the differences outweigh the similarities regarding the relationality of parts in the Chinese source texts vs. the relationality in the English target texts. Specifically, in seven of the twelve paired samples, we observe shifts in the relationality of parts from the source to the target texts. However, we find that no shifts occurred in the rest of the samples. Moreover, we find that the differences in all the relevant elements are diverse. Hence, we detect no unified patterns among these shifts in the relationality of parts, as shown in Table 2 below.

**Table 2:** Shifts in the relationality of parts via translation.

Sample No.	Precipitating event	Evaluation	Coda
1	√		
2	√		
4		√	
11		√	
12		√	
8	√	√	
9	√	√	√

In the seven samples with shifts in their relationality of parts that are shown in Table 2, four paired samples have different precipitating events that start their narratives. Five other paired samples have differences in the evaluations within their narratives. We find a single shift in a coda. Notably, among the samples with shifts, several occur in more than one element. For example, Sample 8 has shifts in both its precipitating event and embedded evaluations from its source to target text. Additionally, the shifts in Sample 9 involve its precipitating events, external evaluations, and coda. Therefore, it cannot be assumed that the relationality of parts in all the samples similarly shifts via translation.

Further, these diverse shifts in the samples’ precipitating events, codas, and/or embedded evaluations and their influences on their corporations’ constitutions of identity are explored below. We find that diverse shifts influence the constitution of these corporate identities to different extents.

For example, in the corporate profile translation of Sample 1, China PetroChemical Corporation, only the precipitating event, that is, the starting point, is shifted from one episode to another via translation. Hence, the corporation’s identity is repositioned in a different context but its focal point remains the same in its TT. Example 6 below allows a comparison of the precipitating events of its ST and TT.

Example 6:

Source Text: 中国石油化工集团公司 (以下简称公司) 的前身是成立于1983年7月的中国石油化工总公司(Sinopec, n.d.)。

Back Translation: The predecessor of China Petrochemical Corporation (hereafter the company) was China Petrochemical Controlling Company, which was established in July 1983.

Target Text: China Petrochemical Corporation (Sinopec Group) is a super-large petroleum and petrochemical enterprise group (Sinopec, n.d.).

**Fig. 4:**
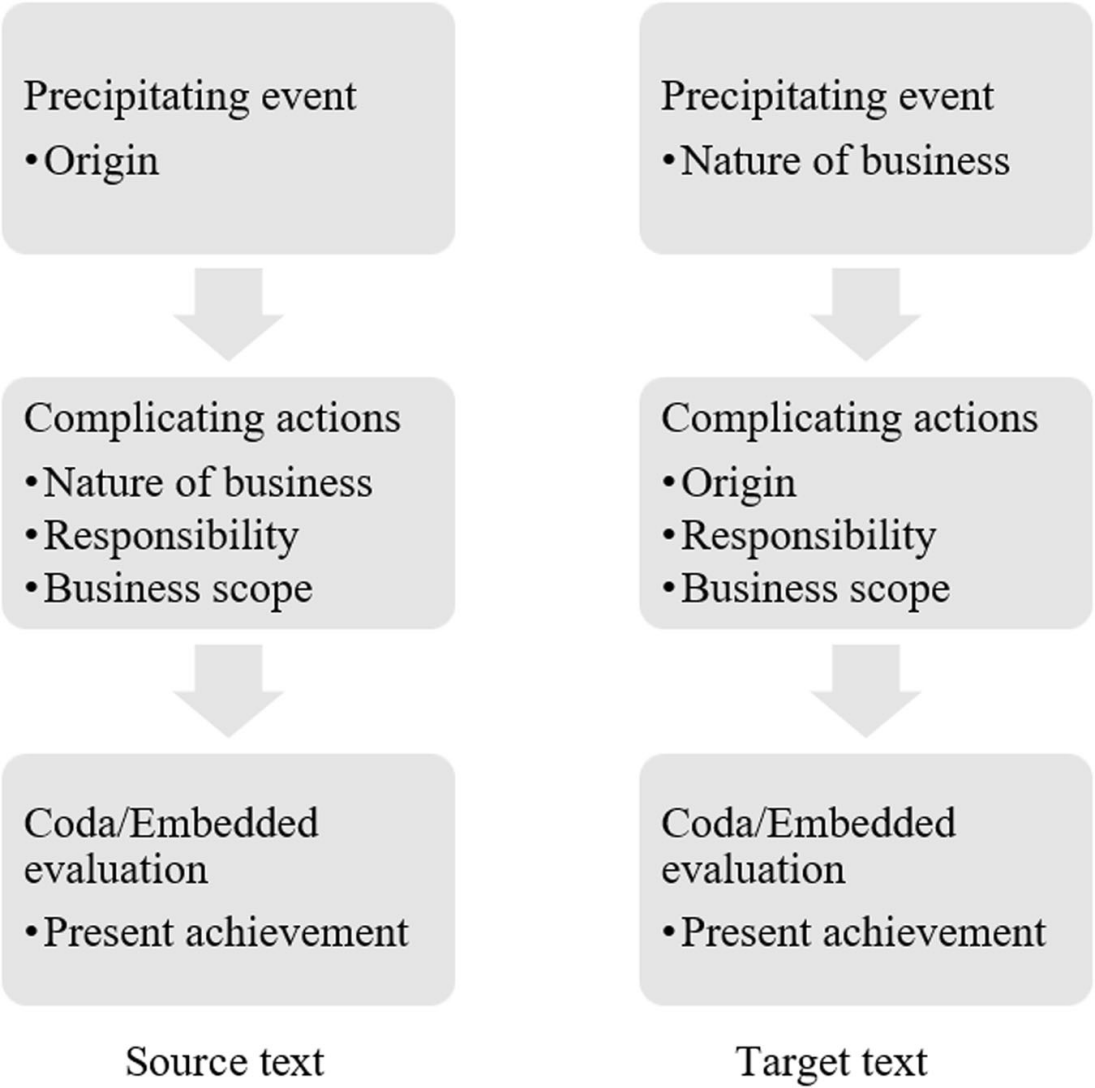
Comparison of the relationality of parts in ST and TT of Sample 1. There is a shift in the precipitating events by permutating the first two episodes while the rest remain the same.

Its ST starts by describing the corporation’s origin as a well-known enterprise with a long history. However, its TT starts with a different precipitating event, the corporation’s present business nature as a super-large enterprise group. The two end with the same coda—an illustration of its present achievement as a world-class enterprise in its industry. The evaluations remain the same as well. This shift is achieved during translation by permutating the first two episodes but not the complicating actions, evaluations, or coda of the ST. As a form of social-cognitive ‘trigger’, precipitating events alone define ‘in part, the forms of collective action’ that take place (Owens, 2013). However, they cannot sufficiently determine the overall relationality of parts in a narrative on their own. Below, Fig. 4 shows how even with different precipitating events, the episodes within these two texts generally remain chronologically related to each other; thus, their narrative focus is on the corporation’s present achievement.

Although it is insufficient to state that only the shift in the precipitating event in Sample 1 leads to its shift in the overall relationality of its parts, the contextual setting of the corporate profile in its TT has been clearly shifted as a consequence. David Waddington and his colleagues have argued that by triggering disorderly events, precipitating events can reveal numerous underlying contextual factors (Owens, 2013). These factors can be either microlevel—among only participants—or macrolevel, involving societal or other broader conditions. In Sample 1, shifting the precipitating event from its state-related origin to the corporation’s business nature entails a shift in what triggers its story. The macrolevel contextual factors in the ST have thus been replaced by microlevel factors in the TT. Specifically, in the ST, a well-known predecessor with a long history is closely related to the corporation; in the TT, the corporation’s own qualification is the driving force. Hence, by shifting the precipitating event, the corporation presents its story in a less externally bounded and more internally bounded contextual setting for its target stakeholders. The corporation’s identity is therefore embedded in a qualitatively different setting following translation.

Similarly, shifts in evaluations alone cannot determine shifts in the overall relationality of parts. Examples of such cases are found in Samples 4, 11, and 12. Rather, by justifying the tellability of different episodes in the ST and TT, evaluations express the critical points of a narrative from the speaker’s perspective (Andrews et al., 2013, p. 32). Evaluations also serve as ‘a continuous and shifting marker of a speaker’s presentation of self’ (Gwyn, 2000, p. 316). In other words, they mark the shifting of the most tellable points of a corporate identity. In this research, our identified shifts in evaluations from source to target text thus indicate shifts in the focal point of a corporate identity, indicating the fluidity of these identities.

For example, the embedded evaluations in Sample 4 are part of the corporate profile of the Aluminium Corporation of China. In its TT, an additional embedded evaluation underscores the corporation’s domestic status as ‘the strongest copper provider in China’ and ‘one of the key, major rare earth companies’ (Aluminium Corporation of China, n.d.). Hence, the corporation’s identity in its TT contains more layers, with more emphasized qualifications, than that in its ST.

However, in some samples, more than one element in the relationality of parts has been shifted during translation. The consequences of these shifts for corporate identity constitution are more evident. For example, in Sample 8, the government’s ownership of the corporation, the precipitating event in its ST, is shifted to its origin in its TT. This is achieved by omitting its ownership information in the beginning of its ST. On the other hand, an embedded evaluation is also added to emphasize the corporation’s progress into an international commerce force in its TT. As a result of these shifts, the contextual macro factor triggering the presentation of the corporation has been transformed into a micro factor. That is, the corporation’s state-owned background has been downplayed, and its identity has been manifested after its origin. Meanwhile, more emphasis on its internalization progress makes the outward facets of its identity stand out in its TT. Overall, the corporation has taken on a more independent and internalized identity in its TT. Accordingly, this sample’s shift in identity constitution is more evident than in those samples with shifts in just one element.

Following the above data analysis of the relationality of parts in the corporate profile translation, we have conducted five in-depth interviews to verify the data analysis results. We then extracted the key themes from the interview transcripts, shown in Table 3.

**Table 3:** Thematic analysis of interview transcripts.

	Key themes
1	Interaction with different parties in translation occurs.
2	Adjustments are made repeatedly in translation.
3	The corporation has its own position in the market.
4	Shifts in the corporation’s images occur in translation.

This thematic analysis shows that in the first place, all the participants regard their work as translation even though they understand the unique features of their work compared to traditional translation. The interplay of different parties occurs in different steps of the translation process, causing repeated adjustments to the translation. The decision to make these adjustments originates mainly with the superiors on a team, especially on more general matters, such as adjusting the profile’s structure in translation, the omission or addition of certain information about the corporation, or the tone of the language. However, the translators have a greater influence concerning specific issues in terms of word choice. For example, a participant described his experience exchanging opinions directly with the corporation’s top management when translating a culturally loaded Chinese word into English.

Second, the analysis shows that each corporation has a precise position in the market and that it defines its language style when translating its corporate profile accordingly. All the participants revealed that they are required to adhere to their corporation’s position when building a desired image in translation.

Nevertheless, different corporations vary in terms of the images they desire to present to foreign stakeholders due to their self-determined position. One participant working for a machinery manufacturing corporation said she was required to be concise and accurate in translation, as the project manager had emphasized that the corporation intends to be considered ‘technical’. Therefore, this translation was almost literal with slight adjustments in the translated profile. Meanwhile, another participant who had translated the profile of a transportation equipment manufacturing corporation repeated the policy-oriented translation strategies several times. He added that this corporation’s management informed him to ‘tell China’s story well’ to foreign markets, saying that it was essential for a state-owned corporation’s corporate communication.

The participants also believed that the adjustments in these corporations’ images were made by carefully considering target stakeholders’ acceptance. One participant attributed such adjustments in translation to the fact that his corporate superiors had studied abroad and knew their target stakeholders well. Another participant cited a survey result concerning European senior target customers’ preferences that was used to justify the translation adjustments on the corporation’s image presentation for the European market.

Although the participants did not discuss the relationality of parts, the findings from these in-depth interviews validate the previous data analysis results. All parties participating in the translation process are thus fully aware that any decisions on the presentation of a corporation to its foreign stakeholders via profile translation must consider its position and desired image, as well as the acceptance of its potential foreign stakeholders.

## Results

Our findings are threefold. First, our results show that there are two patterns in the relationality of parts among all the samples. The first pattern keeps episodes temporally related to each other, with their differences mainly occurring in the continuum of their various meaningful relationships across episodes over time (Kaaristo and Järv, 2012). The first pattern proceeds from a corporation’s origin to its present or future, relating its past, present, and future. However, in the second pattern, which accounts for the relationality of parts in the majority of the samples, episodes are arranged in a causal sequence. Narrative proceeds from business nature to a corporation’s achievements; describing these achievements is proof of success, i.e., the outcome. Therefore, the translations of the corporate profiles of Chinese MNCs are not composed of random events. They follow predominant patterns that organize the parts of their narratives into a whole.

Furthermore, we find that during translation, most samples shift the relationality of parts from their STs to TTs. However, there are no unitary patterns among these shifts. Shifts occur in precipitating events, evaluations and codas, influencing the contextual factors triggering narration and shifting the critical points for presenting a corporation’s story or the outcome of its narration. Moreover, most of the identified shifts are not sufficient to change the overall relationality of parts in these narratives. However, we find that the more elements that are shifted, the more effects there will be on the relationality of parts.

By detecting and analysing shifts in relationality during translations, we have found that the identities in the MNCs’ TTs are diversely positioned in the shifted relational settings of each narrative. Hence, their identities are reconstituted in their TTs to different extents according to their unique position within their relational settings. In some samples, the macro contextual factors that trigger the constitution of identity are shifted to micro factors, invoking different background settings to build a corporation’s identity. In other samples, the focal points in the stories are shifted, emphasizing different facets of a corporate identity. Finally, in very few samples, these reconstitutions are more evident. For example, in Sample 9, as the overall relationship of its episodes is rearranged in translation, the corporation presents a different identity in its TT from that in its ST. Such results reveal their fluidity and uniqueness.

Notably, according to the in-depth interviews, corporations are aware of the corporate profile translation’s influences on their corporate identities. In translation, these corporations’ identities constantly evolve either chronologically or accumulatively and shift to various extents to fulfill their need to present a desired image to their target stakeholders.

## Discussion and conclusion

Corporate profile translation, especially in emerging economies, is a genre offering rich information for narrative studies. Such translations not only possess evolving narrative structures to address the challenges in a market but also constitute identities that are tailored to target stakeholders. In this study, we have conducted a three-step analysis of the relationality of parts and its consequences for corporate identity via corporate profile translation, using twelve Chinese state-owned manufacturing MNCs as our research sample. Our research objectives were to address how the parts of these samples’ narratives are organized, whether they demonstrate any shifts via translation and what, if any, influences of these shifts on the constitution of corporate identities can be identified.

We have found that the formation of parts into a whole narrative in the corporate profiles of China’s manufacturing MNCs follows predominant patterns. Episodes are selected and arranged according to their temporal or causal relationships with each other, thereby constituting dynamic corporate identities. However, there are no unitary patterns for how these corporations shift the relationality of parts when translating their corporate profiles into English. Accordingly, we find diverse shifts in the constitutions of corporate identities for target markets as the result of translation. Therefore, each corporation possesses a unique corporate identity.

This study thus confirms the utility of the relational approach for studying narrative identity constitution, first expressed by Margaret Somers, by integrating it with William Labov’s framework of narrative structure. Our major findings suggest that corporate profiles and their translations are shaped by episodes that are selected due to their tellability and are arranged according to their relationships with each other. This study also confirms that identity constitution is a dynamic process. We have demonstrated that shifted relational networks within MNCs’ narratives drive the shifts in the constitution of their identities from the STs to TTs of their corporate profiles. We have also confirmed that MNCs intentionally use these shifts to design a desired image for their target stakeholders. Nevertheless, this complicates the translation process.

Furthermore, our findings have both practical and theoretical implications. Regarding its theoretical perspective, this study uses a novel approach to examine how parts form whole narratives in the translation of corporate profiles. That is, this study treats narrative as a relational whole and identifies identity constitution as its consequence. Second, our novel findings add to the knowledge on the relationality of parts in corporate profiles and on corporate identity constitution in corporate communications. Third, we highlight the fluidity and uniqueness of corporate identities and test the sociological measures for defining the positions of corporate identities within a relational network.

From a practical perspective, this study could help corporations in emerging economies globalize by finding new ways of strategically presenting themselves to a target market via translation of their corporate profile. Specifically, in difficult economic situations, how and to what extent Chinese MNCs have revised their method of organizing their narrative relational network and planned corporate identity can have implications for other emerging economies. In addition, this study highlights the role that translation plays in corporate communication. Even in highly specific texts, such as corporate profiles, translation can shift the relationality of parts and reconstitute corporate identity to adapt it to a target market. It is a no less difficult task than translation in other fields or generating other forms of corporate communication. Hence, this study can help other corporations better understand the translation process.

There is abundant space for future research based on this study. Since this study uses homogenous sampling, researchers should consider replicating it in a different context. How does the relationality of parts in the profiles of MNCs in other industries function? How do the corporate profiles of MNCs in developed countries form their whole narratives? On the other hand, newer and diverse corporate profiles are emerging that involve multimodal means. Further work investigating the relationality of parts within diverse forms of corporate communications would thus be worthwhile. How can narrative identity theory be applied to other means of corporate communication? How does Labov’s narrative structure apply in a multimodal context? Such findings would be as significant as our results.

## Data Availability

The datasets generated during and/or analysed during the current study are available from the corresponding author on reasonable request.
